# Supplementary material for: Virulence and Infectivity of UC, MD, and L Strains of Infectious Hematopoietic Necrosis Virus (IHNV) in Four Populations of Columbia River Basin Chinook Salmon
Source: Viruses. 2021 Apr 18;13(4):701. doi: 10.3390/v13040701 (PMC8072589; doi:10.3390/v13040701)
Supplement: Supplementary file 1 [file viruses-13-00701-s001.zip › viruses-1138896-supplementary.pdf]

Table S1. Cumulative percent survival (CPS) of triplicate groups of 20 juvenile spring-run and fall-run Chinook salmon of the upper (Up-) and lower (Low-) Columbia River Basin (CRB) exposed to L, UC and MD strains of IHNV. Experimental host populations were exposed to each virus strain at high (2 x 10^5 PFU ml^{-1}) and moderate (2 x 10^3 PFU ml^{-1}) doses by static immersion in 1 L. CPS is reported for individual replicate tanks (a, b, and c) at the end of the 30 d experimental trials.

Columbia River Basin Chinook salmon population	Virus strain	Genogroup, subgroup	High Dose Ind. rep. CPS			Moderate Dose Ind. rep. CPS		
			a	b	c	a	b	c
Up-Spring	FR0031	L	70	30	65	95	95	100
	RB1	UC	90	100	85	100	95	100
	QTS07	MD	95	95	100	100	95	95
Up-Fall	FR0031	L	55	50	65	90	65	70
	RB1	UC	95	90	95	100	100	100
	QTS07	MD	100	100	100	95	100	95
Low-Spring	FR0031	L	80	85	85	100	100	80
	RB1	UC	100	80	100	100	100	80
	QTS07	MD	100	100	100	100	100	100
Low-Fall	FR0031	L	75	65	40	75	95	75
	RB1	UC	100	95	100	95	100	95
	QTS07	MD	100	100	95	85	95	100

Table S2. Parameter estimates, with standard errors (SE), for evaluation of infection status using the full or reduced logistic regression models. The full regression model evaluated infection status against viral strain, exposure dose and host population (model 1). Reduced models omitted either host population (model 2), viral strain (model 3), or both (model 4). The full regression model was favored, as indicated by the lowest observable Akaike's information criterion (AIC).

	Model 1		Model 2		Model 3		Model 4	
	Estimate	SE	Estimate	SE	Estimate	SE	Estimate	SE
β_0	-6.65192	0.73	-6.402	0.66	-6.89648	0.71	-6.6437	0.65
β_{dose}	1.66842	0.16	1.5996	0.16	1.64075	0.16	1.5747	0.15
α_4	0.67208	0.39	-	-	0.66162	0.39	-	-
α_3	-0.07554	0.39	-	-	-0.07434	0.39	-	-
α_2	-0.69391	0.40	-	-	-0.68281	0.39	-	-
γ_m	-0.33893	0.34	-0.3258	0.33	-	-	-	-
γ_u	-0.74167	0.34	-0.7127	0.34	-	-	-	-
AIC#	342.05		348.18		342.85		348.79	

Table S3. Parameter estimates, with standard errors (SE), for evaluation of viral loads using the full or reduced generalized linear models (GLMs). The complex GLM evaluated log viral load outcome against viral strain, exposure dose and host population (model 1). Reduced models omitted either host population (model 2), viral strain (model 3), or both (model 4). The complex GLM was favored, as indicated by the lowest observable Akaike's information criterion (AIC).

	Model 1		Model 2		Model 3		Model 4	
	Estimate	SE	Estimate	SE	Estimate	SE	Estimate	SE
β_0	5.56222	0.54	5.1885	0.54	5.4302	0.53	4.9953	0.53
β_{dose}	0.17506	0.11	0.1992	0.11	0.1692	0.11	0.1954	0.11
α_4	-0.60047	0.23	-	-	-0.6487	0.23	-	-
α_3	-0.47253	0.24	-	-	-0.4854	0.24	-	-
α_2	0.09666	0.26	-	-	0.0344	0.26	-	-
γ_m	-0.19495	0.20	-0.2507	0.20	-	-	-	-
γ_u	-0.4267	0.21	-0.4363	0.21	-	-	-	-
AIC#	459.94		466.55		460.28		466.93	
